# Amino acids in cancer

**DOI:** 10.1038/s12276-020-0375-3

**Published:** 2020-01-24

**Authors:** Elizabeth L. Lieu, Tu Nguyen, Shawn Rhyne, Jiyeon Kim

**Affiliations:** Department of Biochemistry and Molecular Genetics, University of Illinois at Chicago, Chicago, IL USA

**Keywords:** Cancer metabolism, Cell biology

## Abstract

Over 90 years ago, Otto Warburg’s seminal discovery of aerobic glycolysis established metabolic reprogramming as one of the first distinguishing characteristics of cancer^1^. The field of cancer metabolism subsequently revealed additional metabolic alterations in cancer by focusing on central carbon metabolism, including the citric acid cycle and pentose phosphate pathway. Recent reports have, however, uncovered substantial non-carbon metabolism contributions to cancer cell viability and growth. Amino acids, nutrients vital to the survival of all cell types, experience reprogrammed metabolism in cancer. This review outlines the diverse roles of amino acids within the tumor and in the tumor microenvironment. Beyond their role in biosynthesis, they serve as energy sources and help maintain redox balance. In addition, amino acid derivatives contribute to epigenetic regulation and immune responses linked to tumorigenesis and metastasis. Furthermore, in discussing the transporters and transaminases that mediate amino acid uptake and synthesis, we identify potential metabolic liabilities as targets for therapeutic intervention.

## Cancer: How tumors hijack basic building blocks

Cancer changes how the body uses amino acids, the building blocks that all proteins are made of. A better understanding of these changes could lead to new cancer therapies. Cancer was already known to alter the body’s sugar metabolism to feed extra energy to fast-growing tumors. Recent reports have revealed that cancer also rewires amino acid metabolism. Jiyeon Kim at the University of Illinois at Chicago, USA, and co-workers have reviewed how cancer co-opts amino acids. They report that tumors use amino acids as an energy source and antioxidant precursor to balance their production of toxic reactive oxygen species. Amino acids are also instrumental in annotating the epigenetic code to enhance or suppress expression of tumor-related genes. The review illuminates a promising new approach to cancer therapeutics.

## Introduction

The near century of progress^1^ in cancer metabolism continues to impact our understanding of oncology. Each decade has yielded new discoveries and engendered additional avenues of research and approaches to therapy. Classically, cancer metabolism has focused on central carbon metabolism, including glycolysis and the tricarboxylic acid cycle (the citric acid cycle, TCA cycle). However, new studies have shed light on the important role of amino acids in cancer metabolism. Amino acids also serve the essential purposes of redox balance, energetic regulation, biosynthetic support, and homeostatic maintenance. This wide breadth of metabolic activities has made amino acid metabolism increasingly popular in cancer research.

While glucose is a renowned energy source for cancer growth, amino acids are also important fuels supporting cancer development. Glutamine, for example, is largely anaplerotic and relinquishes both amine groups to support the TCA cycle^2^. In addition to glutamine, other amino acids can function as opportunistic fuel sources for cells. Branched-chain amino acids (BCAAs; valine, leucine, and isoleucine) are alternative sources of organic molecules that can also fuel the TCA cycle^3^.

Biosynthetic pathways, like bioenergetic pathways, also rely on various amino acid contributors. Through acetyl-CoA synthesis, lipogenesis can be mediated by catabolism of BCAAs^3^. Nucleotide synthesis, which can be delineated into purine and pyrimidine biosynthesis, is another amino acid-dependent process^4^. Glycine, glutamine, and aspartate serve as carbon and nitrogen donors for purine biosynthesis^5^. Additional sources of carbon for nucleobases as a form of formate include glycine, serine, and methionine, which provide one-carbon units through the methionine–folate cycle^6–8^.

Amino acids produce derivatives that also support cancer growth and metastatic potential. Arginine-derived polyamines alter gene expression by modulating global chromatin structure and cancer cell proliferation^9^. Kynurenine produced from tryptophan induces immunosuppression^10,11^ by binding to and activating the transcription factor aryl hydrocarbon receptor (AhR)^12–14^. This impairs the ability of immune-tolerant dendritic cells (DCs) and regulatory T cells to target and eliminate cancer cells^11,15^.

Cancer cell proliferation results in the accumulation of reactive oxygen species, which can damage macromolecules and ultimately lead to cell death. To counter this, cancer cells are reliant upon the synthesis of glutathione from glutamate, glycine, and cysteine to mediate redox balance^16–18^. While NADPH sourced from glycolysis and the pentose phosphate pathway are commonly identified as the main contributors to redox balance, a significant amount of NADPH is also produced by the folate cycle, which is primarily driven by serine-derived one-carbon units^19^.

In addition to the direct integration of amino acids and their derivatives in metabolic reprogramming processes, amino acids are also fundamental in mediating epigenetic regulation and posttranscriptional modification. For example, DNA and histone methylation are regulated by balanced metabolite levels in the methionine cycle, which are influenced by methionine, serine, and glycine^20,21^. Similarly, histone acetylation events promoting gene expression and cancer progression require acetyl-CoA, which can be derived from BCAAs and lysine^22^. Amino acid-derived acetyl-CoA is also relevant for protein acetylation that can support tumor growth^23,24^.

Transaminases are an important class of enzymes for cancer. By facilitating the interconversion of amino acids, transaminases allow for the exploitation of the diverse functions of amino acids from irregular sources. Aspartate transaminase (AST, or glutamic oxaloacetic transaminase (GOT1 and 2)) is essential for redox balance and growth in pancreatic cancer cells^25^. In colorectal cancer, phosphoserine aminotransferase 1 (PSAT1) levels have been implicated in poor prognosis^26^. The high demand for essential amino acids (EAAs) to support cancer cell growth leads to the upregulation of EAA transporters to meet these requirements, a common feature of many cancers^27–29^.

The wide range of amino acid functions, including amino acid synthesis, breakdown, and transport, has provided numerous targets for the development of drugs. Amino acid-degrading enzymes such as arginase and asparaginase have been shown to exert antitumor effects in preclinical and clinical settings;^30–34^ however, additional pathways involving amino acid transport and synthesis suggest effective therapies. Transporters are attractive therapeutic targets given the availability of various transport inhibitors; this method could be applied to ASCT2 (neutral amino acid transporter B(0), also known as alanine, serine, cysteine transporter 2), which regulates glutamine transport^35^, or xCT (cystine/glutamate antiporter), which provides cysteine for glutathione synthesis^36^. Other avenues of intervention include amino acid synthesis pathways such as targeting the rate-limiting enzyme phosphoglycerate dehydrogenase (PHGDH) of the serine synthesis pathway^37,38^ or indoleamine 2,3-dioxygenase 1 (IDO1), which generates kynurenine from tryptophan^39,40^. Overall, multiple factors involved in the regulation and synthesis of amino acids could be subject to potential therapeutic intervention.

The principal focus of this review is the specific relationship between amino acids and cancer metabolism. Relevant work detailing nonessential amino acid (NEAA) metabolism, transporters, and transaminase in cancer is covered in several reviews and in the cited articles^2,6,41–44^. These in combination with recently published studies supplement the discussion of the roles of amino acids, which include bioenergetic formation, biosynthetic activity, and resistance to metabolic stress. This review also discusses the regulation of amino acid metabolism in cancer, therapies that are under investigation, and aspects of amino acid metabolism relevant to inhibition with a specific focus on transaminases and amino acid transporters.

## Amino acids as alternative fuels

**Fig. 1:**
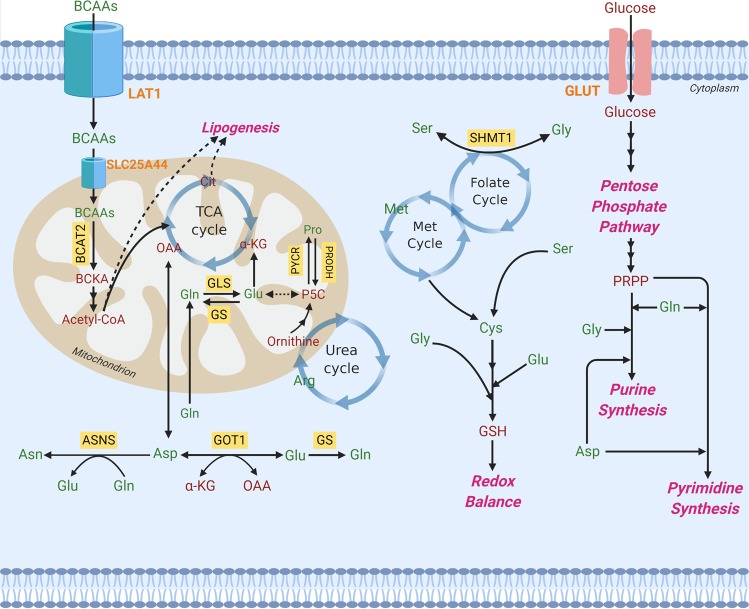
Amino acids in metabolic pathways. Metabolic reprogramming is a staple of cancer cell growth and proliferation. Both essential and nonessential amino acids (EAAs and NEAAs) support altered metabolism by serving as energy sources, biosynthetic molecules, and mediators of redox balance. Amino acids produce metabolic intermediates, such as acetyl-CoA, that sustain energy synthesis through the citric acid cycle. Amino acids also provide building blocks for nucleotide synthesis and lipogenesis that are critical to a cell’s ability to grow and develop. To circumvent the effects of oxidative stress, amino acids can regulate redox balance through their production of glutathione. Furthermore, EAA catabolism contributes to the generation of NEAAs through chemical reactions, including those mediated by transaminases. Amino acids are in green, and other metabolites are in red. Orange represents transporters. Yellow boxes signify enzymes. SHMT1 serine hydroxymethyltransferase, cytosolic, BCAT branched-chain amino acid transaminase, mitochondrial, BCAA branched-chain amino acid (valine, leucine, isoleucine), BCKA branched-chain ketoacid, GOT1 aspartate transaminase, cytosolic (AST), GLS glutaminase, GS glutamine synthetase (cytosolic and mitochondrial), ASNS asparagine synthetase, PRODH pyrroline-5-carboxylate dehydrogenase, PYCR pyrroline-5-carboxylate reductase, P5C pyrroline-5-carboxylate, GSH glutathione, Gly glycine, Ser serine, Met methionine, Met cycle methionine cycle, Gln glutamine, Cys cysteine, Glu glutamate, Asp aspartate, Pro proline, Asn asparagine, Arg arginine, PRPP phosphoribosyl pyrophosphate, acetyl-coA acetyl-coenzyme A, α-KG alpha-ketoglutaric acid, OAA oxaloacetic acid, LAT1 large-neutral amino acid transporter 1, SLC25A44 solute carrier family 25 member 44, GLUT glucose transporter, TCA cycle the tricarboxylic acid (also known as the citric acid cycle).

In cancer cells, glutamine is the major amino acid that serves as an anaplerosis metabolite and drives the tricarboxylic acid (TCA) cycle to sustain mitochondrial ATP production. Anaplerotic metabolism of glutamine generates α-ketoglutarate (α-KG) and subsequently oxaloacetate (OAA) and fuels the TCA cycle through a series of biochemical reactions termed glutaminolysis^2^ (Figs. 1, 4b). Under glucose-deprived conditions, glutamine-derived fumarate, malate, and citrate are significantly increased^45^. Similarly, under hypoxia or in cancer cells with mitochondrial dysfunctions, the direction of metabolic flow and utilization of glutamine is drastically changed. In such conditions, α-KG from glutamine can undergo reductive carboxylation to generate isocitrate, which is then converted into citrate^46,47^. Upon glutamine deprivation, asparagine plays a critical role in suppressing apoptotic cell death^48,49^. The positive regulator that transduces a signal from glutamine depletion to apoptosis is citrate synthase (CS)^48^. Under normal circumstances, CS condenses OAA from glutamine with acetyl-CoA to maintain the TCA cycle function. Silencing CS leads to enhanced OAA diversion from the TCA cycle to aspartate and asparagine biosynthesis, protecting cells from glutamine depletion-mediated apoptosis^48^. Exogenous asparagine completely restored cell survival under glutamine-depleted conditions, whereas silencing asparagine synthetase (ASNS) led to apoptosis even in the presence of glutamine. These observations highlight the potential importance of ASNS during tumor cell accumulation and progression (when glutamine availability is limited). Indeed, ASNS expression is associated with poor prognosis in brain tumors, such as glioma and neuroblastoma^48^.

In addition to glutamine, cancer cells also utilize other amino acids as “alternative fuels” to compete for energy with various cells in tumor stroma and to optimize nutrient utilization during the evolution of the tumor^41^. This contrasts with prevailing views that transforming mutations impose a rigid dependence on specific nutrients, such as glucose^50^. In human pancreatic ductal adenocarcinoma (PDAC), elevation of circulating BCAAs (leucine, isoleucine, and valine) is an early event of tumor development^51^. Given that 95% of PDAC is KRAS-driven and that KRAS plays an important role in scavenging nutrients^52,53^, elevated plasma levels of BCAAs may indicate a prominent role of protein breakdown in KRAS-mutant PDAC. BCAAs can provide an anaplerotic substrate in some tissues (Fig. 1)^3^. Elevated BCAAs in the plasma of patients with pancreatic cancers^51^ can potentially be converted into acetyl-CoA and other organic molecules that also enter the TCA cycle. Among BCAAs, leucine is important for melanoma cell survival^54^. Hyperactivation of the RAS–MEK pathway in melanoma renders cancer cells dependent on leucine, and leucine deprivation in melanoma cells fails to appropriately activate autophagy, subsequently leading to apoptotic death^54^. Although not in a cancer setting, threonine can yield acetyl-CoA to feed the TCA cycle. Threonine catabolism mediated by threonine dehydrogenase (TDH) produces glycine and acetyl-CoA, which is then utilized to sustain mitochondrial ATP production in mouse embryonic stem cells (eSCs)^55^.

## Amino acids as biosynthetic materials

Enhanced biosynthetic activities are an essential feature of metabolic reprogramming in cancer: they support cells to produce the macromolecules required for DNA replication, cell division, and subsequent tumor growth. Biosynthetic pathways or anabolic pathways convert simple metabolites (e.g., sugars and amino acids) to complex molecules through ATP-dependent processes. Amino acids are involved in synthesizing three primary macromolecules: proteins, lipids, and nucleic acids (Fig. 1). All 20 canonical amino acids are proteinogenic, but only a subset of amino acids is involved in nonessential amino acid (NEAA) synthesis (e.g., glutamine, glutamate, methionine, and phenylalanine). Among the amino acids involved in NEAA synthesis, glutamine supplies nitrogen for synthesizing the amide group of asparagine. It also contributes to the synthesis of several amino acids through its catabolism to glutamate. Glutamine is converted to glutamate, mainly by glutaminase (GLS) activity (Figs. 1, 4b)^42^. Glutamate can then be further converted to α-KG and other amino acids, such as alanine, aspartate, and phosphoserine, by aminotransferase reactions (Figs. 1, 4b)^43^.

**Fig. 2:**
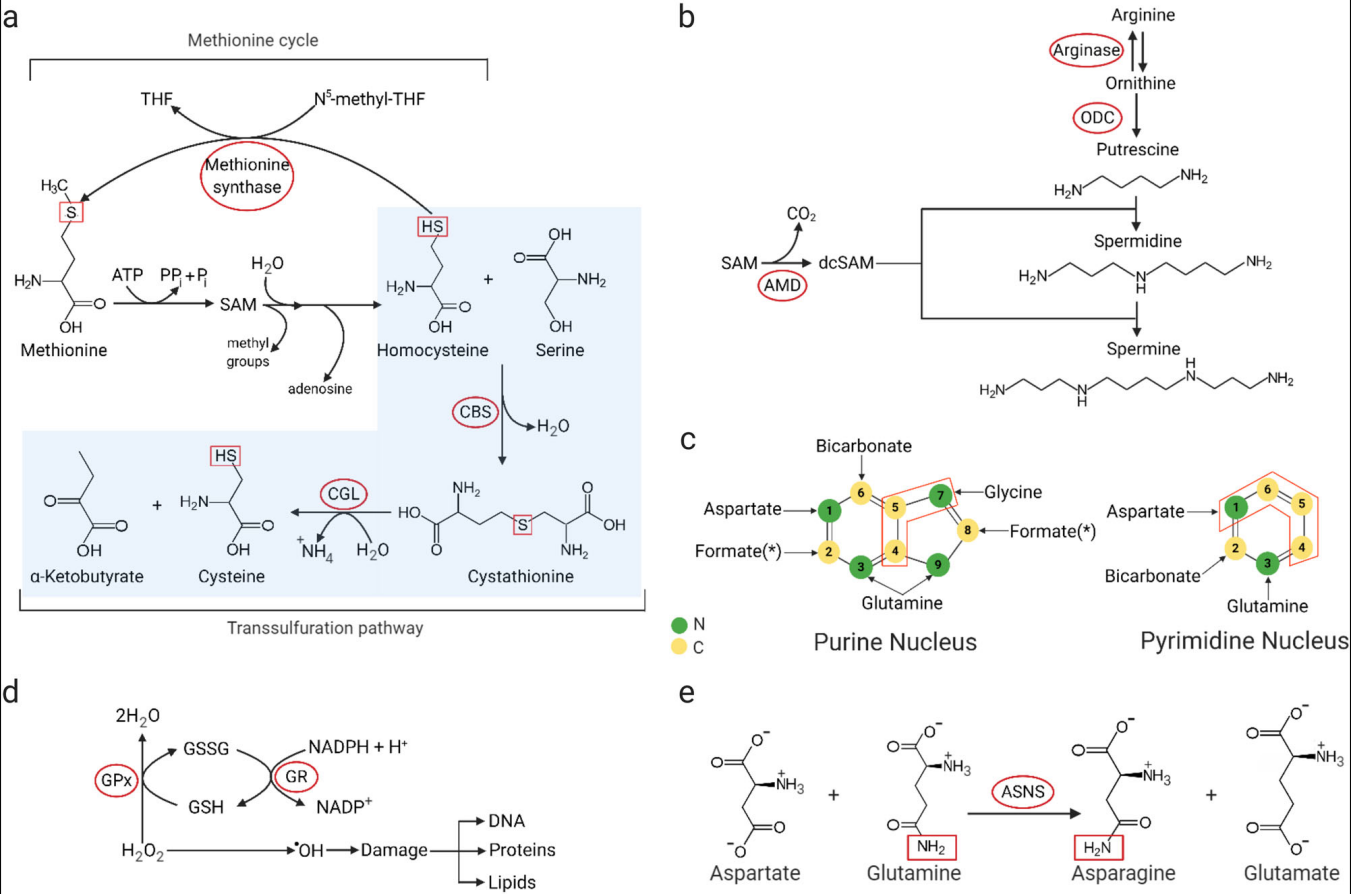
Biochemical reactions in amino acid metabolism. **a** Reverse-transsulfuration pathway: Cysteine can be produced from methionine through the reverse-transsulfuration pathway. This pathway is a combination of the methionine cycle and transsulfuration pathway. Homocysteine, the intermediate of the first step in the transsulfuration pathway, is generated from the methionine cycle. Serine condenses with homocysteine, producing cystathionine. Cystathionine is then converted to cysteine and alpha-ketobutyrate by CGL. Key enzymes are in red circle. THF tetrahydrofolate, CBS cystathionine β-synthase, SAM S-adenosylmethionine, CGL cystathionine γ-lyase. **b** Polyamine synthesis: Polyamines (putrescine, spermine, and spermidine) are synthesized from the amino acid arginine, and are converted from one to another (in the order of putrescine to spermidine to spermine). SAM, as the precursor of dcSAM, is the major donor for constructing polyamine structures. Key enzymes are in red circle. ODC ornithine decarboxylase, AMD S-adenosylmethionine decarboxylase, SAM S-adenosylmethionine, dcSAM decarboxylated S-adenosylmethionine. **c** Nitrogen and carbon source for nucleic acids: Aspartate, glycine, and glutamine provide nitrogen, and glycine and one-carbon units from the folate cycle (as a form of formate) provide carbon for purines. Glycine is formate’s indirect precursor through one-carbon metabolism, providing formate for biochemical reactions in purine biosynthesis. Aspartate and glutamine are the main amino acids involved in pyrimidine synthesis. Carbon (C) is in yellow, and nitrogen (N) is in green. **d** GSH and NADPH as antioxidants: Reactive oxygen species (ROS) bind and damage cellular macromolecules. The oxidation of NADPH and GSH allows ROS to be reduced to an inactive state. GSH reduces hydrogen peroxide to water and becomes oxidized to GSSG by GPX. Oxidized glutathione (GSSG) is then reduced back to GSH by GR in the presence of NADPH. Enzymes are shown in red circles. GPX glutathione peroxidase, GR glutathione reductase, GSH reduced glutathione, GSSG oxidized glutathione, NADPH reduced nicotinamide adenine dinucleotide phosphate, NADP+ oxidized nicotinamide adenine dinucleotide phosphate. **e** Amidation reaction for asparagine synthesis: Asparagine is synthesized by an amidotransferase reaction, catalyzed by asparagine synthetase (ASNS). The conserved amide group nitrogen is in a red box, while the enzyme is in a red circle.

Unlike the aforementioned glutamine-derived amino acids, which need glutamate as a nitrogen donor, asparagine requires glutamine for de novo synthesis (Figs. 1, 2e, 4b). Glutamine is a substrate for asparagine synthetase (ASNS), providing amide nitrogen to aspartate to produce asparagine. Arginine is another amino acid used to synthesize nonessential amino acids. It can serve as the precursor to proline or as an additional source of glutamate, both via the intermediacy of 1-pyrroline-5-carboxylate (P5C) (Fig. 1)^56^. 1-Pyrroline-5-carboxylate reductase (PYCR) converts P5C into proline, and 1-pyrroline-5-carboxylate dehydrogenase (P5CDH encoded by *ALDH4A1*) catalyzes the conversion of P5C into glutamate^57^. Serine and glycine are closely related and can be interconverted by serine hydroxymethyltransferase (SHMT1 and 2) (Fig. 1)^58^. SHMT is one of the key enzymes in folate-mediated one-carbon metabolism. One-carbon metabolism encompasses both the folate and methionine cycle and provides methyl groups for the one-carbon pools that are required for de novo nucleotide biosynthesis and DNA methylation^59^. Tetrahydrofolate (THF) serves as a universal one-carbon acceptor and can accept one-carbon from the conversion of serine to glycine (folate cycle), the oxidation of glycine to CO_2_ and NH_3_ by the glycine cleavage system (GCS)^6^ or methionine to homocysteine conversion (methionine cycle)^59^. One-carbon bound THF exists in different oxidation states (5,10-methylene tetrahydrofolate (5,10-meTHF), 5-methyl-THF, formate (10-formyl THF)), and supports distinct biosynthetic functions, for example, 5,10-meTHF for pyrimidine biosynthesis and 5-methyl-THF for purine biosynthesis^59^.

The sulfur containing amino acid methionine can provide cysteine by the reverse-transsulfuration pathway (Fig. 2a). Homocysteine generated from the methionine cycle is condensed with serine to become cystathionine by cystathionine β-synthase (CBS), and is then transformed to cysteine by cystathionine γ-lyase (CGL) (Fig. 2a). Methionine to cysteine conversion is connected to serine–glycine interconversion because the methionine cycle is part of one-carbon metabolism coupled with the folate cycle, where SHMT catalyzes serine–glycine synthesis (Fig. 1)^6^. While asparagine is not directly involved in NEAA biosynthesis, it acts as an amino acid exchange factor and promotes amino acid uptake, preferentially of serine/threonine and nonpolar amino acids^60^, and can potentially support further building block synthesis.

Amino acids provide both carbon and nitrogen for nucleic acid synthesis (Fig. 2c). Purine biosynthesis requires formate, bicarbonate, and three amino acids: aspartate, glycine, and glutamine. While glutamine and aspartate act as the nitrogen source for both nucleobases (N1 from aspartate and N3 and N9 from glutamine) and the amino group of purines (glutamine for adenine and aspartate for guanine), glycine can contribute to purine biosynthesis in two ways: by direct incorporation into the purine backbone (C4, C5, and N7) or by producing one-carbon units for biochemical reactions involved in purine biosynthesis (C2 and C8) (Fig. 2c)^5^. The critical carrier of one-carbon units in the latter process is 5,10-meTHF. 5,10-meTHF is further converted to formate (10-formyl THF), contributing C2 and C8 carbons to the nucleobase^4,7,61^. Pyrimidine biosynthesis is simpler than that of purine. In contrast to purines that are synthesized as ribonucleotides rather than as nucleobases, pyrimidines are synthesized first as nucleobases and then conjugated to phosphoribosyl pyrophosphate (PRPP) to yield the corresponding ribonucleotide. The pyrimidine ring is derived from glutamine, aspartate, and bicarbonate. For pyrimidine synthesis, aspartate acts as both carbon and nitrogen donors (N1, C4, C5, and C6), whereas glutamine contributes to N3 of the nucleobase and amino group of cytosine (Fig. 2c)^4^. The one-carbon unit derived from serine to glycine conversion is required for thymidylate synthesis. 5,10-meTHF serves as a one-carbon donor to transfer a methyl group to deoxyuridine monophosphate (dUMP) and produce deoxythymidine monophosphate (dTMP), a reaction catalyzed by thymidylate synthase (TS)^7,8^.

In addition to their primary role in the biosynthesis of nitrogenous metabolites, amino acids can supply carbon atoms for lipid biosynthesis. Under hypoxia, glutamine contributes to the acetyl-CoA pools needed for lipogenesis by being converted into pyruvate that reenters the TCA cycle^46,62^. BCAAs can also contribute to lipogenesis. In differentiated adipocytes, BCAA catabolic flux increases, and BCAA-derived acetyl-CoA accounts for approximately 30% of the lipogenic acetyl-CoA pools^3,63^. Essential amino acids (EAAs) act not only as carbon donors, but their ratio can also regulate lipogenesis by impacting lipogenic gene expression^64^. In bovine mammary epithelial cells, the “optimal” amino acid (AA) ratio (OPAA = Lys:Met 2.9:1; Thr:Phe 1.05:1; Lys:Thr 1.8:1; Lys:His 2.38:1; Lys:Val 1.23:1) upregulates lipogenic gene expression and alters the expression of key miRNAs involved in the control of lipogenic balance, implying a potentially important role of EAA ratios in lipid synthesis^64^. It would be interesting to see if this is conserved in other species as well as in cancer.

## Tumor-associated amino acid derivatives

Amino acid catabolism produces metabolic intermediates affecting tumor cell growth and survival. Polyamines (putrescine, spermine, and spermidine) might be the best-known metabolites to promote tumor proliferation and aggressiveness^65^. Polyamine synthesis starts from arginine conversion to ornithine through the action of arginase, which is then decarboxylated by the rate-limiting step enzyme, ornithine decarboxylase (ODC), to produce putrescine (Fig. 2b). Decarboxylated S-adenosylmethionine, catalyzed by S-adenosylmethionine decarboxylase (AMD)^66^, then donates its propyl amine moiety to putrescine and spermidine for the formation of spermidine and spermine, respectively (Fig. 2b)^67^. Elevated polyamine levels have been observed in patients with cancer. Polyamines and their metabolites in urine and plasma can be useful in both cancer diagnosis and as markers of tumor progression in lung and liver cancers^68,69^. Polyamines affect numerous processes in tumorigenesis, in part by regulating specific gene expression transcriptionally. As charged cations at physiological pH, polyamines can associate with nucleic acids^70^, which in turn can affect global chromatin structure^71^ as well as specific DNA–protein interactions^72^, leading to impacts on gene transcription. Posttranscriptional aspects of polyamine-mediated gene regulation are associated with the eukaryotic translation initiation factor 5A (eIF5A), whose expression/function is strongly correlated with unfavorable prognostic implications for several cancers^73–75^. The spermidine-derived amino acid hypusine, a unique eIF5A posttranslational modification of lysine residue 50, is essential for eIF5A functions^76^. Polyamines also exist as a polyamine-RNA complex^77^. Polyamine binding to RNA leads to structural changes, which stimulate and increase the efficiency of protein synthesis.

Nitric oxide (NO) is another metabolic consequence of arginine catabolism. Depending on its timing, location, and concentration, it has both tumor-suppressive and tumor-promoting effects^78^. It promotes tumor growth through multiple mechanisms, including increasing angiogenesis and limiting the host immune response against tumors. It can, however, also act as a tumor-suppressive molecule by activating caspases and upregulating tumor suppressor p53^78^. Thus, a better understanding of NO biology and further validation with molecular and clinical studies are necessary to develop NO-based strategies for cancer prevention and treatment.

**Fig. 3:**
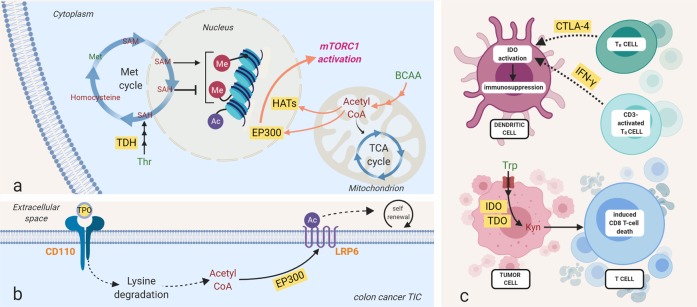
Amino acids contribute to epigenetic and protein regulation and immunosuppression. **a** Amino acids provide metabolic intermediates for epigenetic regulation. One-carbon units from the methionine (shown here) and folate cycle serve as a methyl donor for DNA and histone methyltransferases, while acetyl-CoA from BCAAs and leucine can be utilized for histone acetylation. **b** Amino acid-derived acetyl-CoA is also involved in protein acetylation modification; a thrombopoietin (TPO)-responsive homodimeric receptor, CD110, activates lysine catabolism, which generates acetyl-CoA for LRP6 (a Wnt signaling protein) acetylation and promotes the self-renewal of tumor-initiating cells of colorectal cancer^24^. **c** Elevated kynurenine (Kyn) levels originating from tryptophan via the enzymes tryptophan 2,3-dioxygenase (TDO) and indoleamine 2,3-dioxygenase (IDO) have been shown in several cancers, including Hodgkin lymphoma, lung cancer, and ovarian cancer. Kynurenine promotes tumor cell survival by both inducing T-cell death and inducing immune tolerance in dendritic cells (DCs). Methylation and acetylation are represented by red Me and blue Ac circles, respectively. Histone methylation and acetylation are represented by curved lines. DNA methylation is represented by a straight line. Amino acids are in green, and other metabolites are in red. Orange represents receptors. Yellow boxes signify proteins. SAM S-adenosylmethionine, SAH S-adenosyl homocysteine, Met methionine, Thr threonine, BCAAs branched-chain amino acids, Leu leucine, Lys lysine, Acetyl-CoA acetyl-coenzyme A, Trp tryptophan, Kyn kynurenine, IFN-γ interferon gamma, mTORC1 mammalian target of rapamycin complex 1, TDH threonine dehydrogenase, EP300 histone acetyltransferase p300, HAT histone acetyltransferase, CD110 myeloproliferative leukemia protein (thrombopoietin receptor), TPO thrombopoietin, IDO indoleamine 2,3-dioxygenase, TDO tryptophan 2,3-dioxygenase, CTLA-4 cytotoxic T-lymphocyte-associated protein 4, T_R_ cell, regulatory T cell.

Kynurenine is a tumor-associated metabolite that is catabolized from tryptophan by tryptophan 2,3-dioxygenase (TDO) and indoleamine 2,3-dioxygenase (IDO)^79^. An increased kynurenine to tryptophan ratio has been observed in various tumors, including Hodgkin lymphoma, lung cancer, and ovarian cancer^80–82^. The important role of kynurenine is linked to its ability to suppress antitumor immune responses. Kynurenine secreted from tumors induces cytotoxic CD8 T-cell death, enhancing immune evasion during metastasis^10^. Importantly, kynurenine-mediated immunosuppression is not limited to cross talk between cancer cells and immune cells (Fig. 3c). Communication within the different immune cells also regulates their kynurenine synthesis^11,15^. Regulatory T (T_R_) cells activate IDO in DCs, priming DCs for tolerance induction through CTLA-4 (resting T_R_ cells) or IFN-γ (CD3-activated T_R_ cells) (Fig. 3c)^11,15^. As immunometabolic adjuvants to widen therapeutic windows, IDO inhibitors may leverage not only immuno-oncology modalities but also conventional chemotherapy and/or radiotherapy.

The proline downstream metabolite, 1-pyrroline-5-carboxylic acid (P5C, also known as hydroxyproline), has implications for tumor aggressiveness. Hydroxyproline provides a direct bridge between the TCA (glutamate and α-KG) and urea cycle (ornithine) (Fig. 1)^83^. It is correlated with hepatocellular carcinoma (HCC) clinical pathogenesis^84^. Mechanistically, hypoxic microenvironments activate proline metabolism, resulting in the accumulation of hydroxyproline that promotes HCC tumor progression and induces sorafenib resistance by modulating hypoxia-inducible factor 1-alpha (HIF1α)^84^.

## Amino acids for redox balance

Cancer cells inevitably produce high levels of reactive oxygen species (ROS) due to their highly proliferative nature. ROS are intracellular chemical species containing oxygen and include the superoxide anion (O_2_^−^), hydrogen peroxide (H_2_O_2_), and the hydroxyl radical (OH·). Oxygen radicals produced from ROS can covalently bind to and oxidize macromolecules (lipids, proteins, and DNA), leading to cellular damage (Fig. 2d). Consequently, increased ROS production requires coupling with increased antioxidant defense production to protect cancer cells from ROS-mediated demise; thus, cancer cells allocate significant energy to maintain their intracellular redox balance. Key metabolic players that control the redox state are reduced nicotinamide adenine dinucleotide phosphate (NADPH) and reduced glutathione (GSH) (Fig. 2d). NADPH is a cofactor that not only provides reducing power for macromolecule biosynthesis, but also functions as an antioxidant by acting as a hydride (hydrogen anion) donor in various enzymatic processes, including reduction of glutathione disulfide (GSSG) back to GSH (Fig. 2d). GSH is an essential thiol antioxidant and plays a key role in controlling the redox state of all subcellular compartments^85^. GSH reduces hydrogen peroxide to water and becomes oxidized to GSSG in the presence of GSH peroxidase (GPX) (Fig. 2d). Oxidized glutathione (GSSG) is then reduced back to GSH by glutathione reductase (GR) and NADPH. Amino acids are the main elements for GSH synthesis and NADPH generation. Glutathione synthesis requires three amino acids: glutamate, glycine, and cysteine. Among them, cysteine is the key element because of its thiol group (R-SH), which has redox properties. Inhibiting cysteine uptake reduces viability due to cell death caused by uncontrolled oxidative stresses^17,18,86^. Cysteine can be imported into cells either directly or in its oxidized form, cystine, through the cystine/glutamate antiporter system x_c_^−^ (xCT). Once in the cell, cystine is immediately reduced to cysteine either by intracellular GSH via the formation of a mixed disulfide intermediate or by thioredoxin reductase 1 (TRR1)^87^. The rate-limiting step of glutathione synthesis is the ATP-dependent condensation of cysteine and glutamate to form the dipeptide γ-glutamylcysteine by glutamate cysteine ligase (GCL)^88^. Glycine is then added to the C-terminal of γ-glutamylcysteine to produce glutathione.

The reducing equivalent NADPH is required to maintain multiple antioxidant defense systems. It has been generally accepted that the main route to produce cellular NADPH is glucose via the pentose phosphate pathway (PPP). However, a growing body of research has uncovered that serine-driven one-carbon metabolism via the folate cycle contributes nearly as much to NADPH production as the PPP and malic enzymes in proliferating cells^19^. In the folate cycle, serine to glycine conversion produces 5,10-meTHF, which is oxidized to 10-formyl-tetrahydrofolate (formate/10-formyl THF)^6^. The latter reaction is coupled to the reduction of NADP+ to NADPH. SHMT-mediated serine catabolism, especially mitochondrial SHMT2 reaction, is critical for redox regulation under hypoxia^89^. SHMT2 is induced by hypoxic stress through HIF1α and is involved in maintaining the cellular NADPH/NADP+ ratio^89^. Serine is also involved in GSH synthesis via the folate cycle^90^. In addition to NADPH production, the folate cycle contributes to the production of GSH by intersecting with the methionine cycle^6^. Thus, it is not surprising that serine depletion results in glutathione reduction^91^. Indeed, activation of serine synthesis is now well identified as a bypass of glycolytic flux contributing to GSH synthesis^92,93^. Considering the importance of amino acids in redox homeostasis, the transport and internal synthesis pathways for cysteine, serine, glutamine, and to some extent glycine would be legitimate targets for the development of novel redox-based therapeutics.

## Amino acids as epigenetic and posttranscriptional regulators

Epigenetic alterations are heritable features that affect cellular phenotypes by modifying gene expression independent of the DNA sequence^94,95^. Epigenetic control of gene expression is fine-tuned by a balance between enzymes that “write” regulatory marks onto DNA and histone proteins (e.g., DNA- and histone methyltransferases and histone acetyltransferases) and other enzymes that “erase” these same marks (e.g., histone deacetylases, histone demethylases, and DNA demethylases)^96^. DNA methylation is mediated by DNA methyltransferases (DNMTs), which catalyze the covalent addition of a methyl group to cytosine to form 5-methylcytosine in the context of CpG dinucleotides^97^. In general, methylation of CpG islands in promoter regions inhibits transcription. Global DNA hypomethylation and hypermethylation of promoters of tumor suppressor genes and homeobox genes is a ubiquitous feature of the cancer genome of DNA^98–100^. Histone methyltransferases (HMTs) catalyze the transfer of mono- to tri-methyl groups to lysine and arginine residues of histone proteins^101^. Histone methylation can be involved in either activation or repression of gene expression, depending on which residue is modified and how many methyl groups are incorporated. Methionine, by serving as the methyl group donor for methylation, is the major amino acid that contributes to epigenetic regulation (Fig. 3a). Both DNMTs and HMTs utilize S-adenosylmethionine (SAM) as a methyl donor. SAM is generated in the methionine cycle by methionine adenosyltransferase (MAT) using methionine and ATP as substrates^102^. After donating a methyl group, SAM becomes S-adenosyl-homocysteine (SAH), which inhibits both DNMTs and HMTs (Fig. 3a). Consequently, alterations in the SAM/SAH ratio regulate these methyltransferases’ activity^103^. The importance of SAM in tumor survival has been observed in various studies where cancer cells’ unique requirement for methionine is based on SAM dependence, not methionine dependence^104,105^. The enhanced methionine cycle leads to an excess supply of SAM. This in turn causes DNA hypermethylation and inappropriate gene silencing as well as aberrant histone methylation^20,106^ and enhanced tumor growth. Threonine catabolism mediated by threonine dehydrogenase (TDH) can also provide a precursor for SAM (Fig. 3a)^107^. Glycine from the TDH reaction facilitates one-carbon metabolism via the glycine cleavage system. Threonine depletion or TDH deletion reduces SAM levels and decreases trimethylation of histone H3 on lysine residue 4 (H3Kme3)^107^. Although TDH enzyme function is lost in humans, it certainly suggests that human tumorigenesis can be related to a dysregulation in this metabolic pathway (e.g., upregulation of threonine uptake).

Histone acetylation increases DNA accessibility by reducing the attractive electronic interactions between histones and DNA, thereby supporting gene expression. It is regulated by opposing actions of histone acetyltransferases (HATs) and histone deacetylases (HDACs) that catalyze the addition and removal of the acetyl group on lysine residues, respectively^108^. Acetyl-CoA is an essential metabolic intermediate that regulates acetylation status. HATs utilize the acetyl group of acetyl-CoA to form ε-N-acetyl lysine, and evidence indicates that both acetyl-CoA abundance and the ratio of acetyl-CoA to coenzyme A regulate histone acetylation in cancer^109^.

BCAAs (leucine, isoleucine, and valine) and lysine are catabolized to acetyl-CoA that can potentially be utilized by HATs^22^. Importantly, the acetyl-CoA pools from amino acids also modulate protein acetylation and can promote tumor growth. Leucine provides acetyl-CoA to the EP300 acetyltransferase, which mediates inhibitory acetylation of the mTORC1 regulator Raptor at K1097 and leads to mTORC1 activation (Fig. 3a)^23^. Lysine-derived acetyl-CoA plays an important role in the self-renewal of tumor-initiating cells (TICs) of colorectal cancer (Fig. 3b)^24^. CD110, a thrombopoietin (TPO)-responsive homodimeric receptor, is a key molecule expressed in colon TICs that drives liver metastasis^24^. Upon TPO binding, CD110 activates lysine degradation, which generates acetyl-CoA for acetylation of LRP6 (Fig. 3b), a coreceptor of Wnt signaling crucial for self-renewal of colon TICs^110^, in an EP300-dependent manner. Acetylation of LRP6 stimulates LRP6 activity and self-renewal of CD110+ colon TICs. Collectively, amino acid catabolism involves both epigenetic regulation and posttranslational modification of key proteins in the survival and proliferation signaling pathways, impacting tumor aggressiveness.

## Regulation of amino acid metabolic enzymes and transporters

**Fig. 4:**
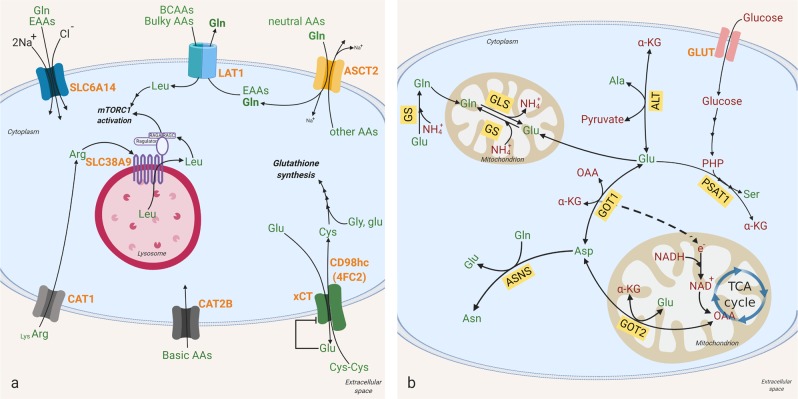
Amino acid transporters and transaminases. **a**, **b** Cells can either import amino acids from the extracellular environment or synthesize them inside the cell. Amino acid transporters are required to transfer them across the plasma membrane (shown in **a**), and transaminases are required to generate amino acids from sugar precursors and nitrogen from other amino acids (shown in **b**). Amino acid transporters and transaminases contribute to metabolic reprogramming in cancer through manipulation of the amino acid pool inside and outside cancer cells. Their impact on the tumor microenvironment is associated with tumor aggressiveness; some induce tumor cell growth and expansion through activation of signaling pathways (e.g., mTORC1 activation) or inactivation of tumor suppressors, while others result in apoptotic cell death. Amino acids are in green, and other metabolites are in red. Bright red represents transporters. Yellow boxes signify enzymes. The relative sizes of Lys and Arg represent the tendency to be transferred across CAT1 in a cancer setting. SLC6A14 solute carrier family 6 member 14, LAT1 large-neutral amino acid transporter 1, ASCT2 alanine, serine, cysteine-preferring transporter 2, xCT/CD98hc (4FC2) cystine/glutamate heterodimeric antiporter, CAT2B cationic amino acid transporter 2B, CAT1 cationic amino acid transporter 1, GLS glutaminase, GS glutamine synthetase, PSAT1 phosphoserine aminotransferase 1, AST aspartate aminotransferase, also known as glutamic oxaloacetic transaminase (GOT). GOT1 is in the cytosol and GOT2 in mitochondria, ASNS asparagine synthetase, ALT alanine aminotransferase, EAA essential amino acid, Na^+^, sodium ion, Cl^−^ chloride ion, Gln glutamine, Leu leucine, AA amino acid, Gly glycine, Glu glutamic acid, Cys cysteine, Cys cystine, Lys, lysine, NH_4_^+^ ammonium ion, Asn asparagine, Ala alanine, Asp aspartic acid, Ser serine, PHP 3-phosphohydroxypyruvate, α-KG alpha-ketoglutarate, e^−^ electron, NADPH reduced nicotinamide adenine dinucleotide phosphate, NAD^+^ oxidized NADPH, OAA oxaloacetate.

Cancer cells reprogram metabolism in ways that can meet their increased demand for amino acids^37,111–113^. Amino acids can be taken up from the extracellular environment or, in the case of NEAAs, be synthesized by various reactions, including transaminase reactions. Cancer cells display enhanced amino acid uptake^114,115^. Because of their hydrophilic nature, amino acids require a transporter system to cross the plasma membrane. Most amino acid transporters recognize more than one amino acid as a substrate. Among the transporters, SLC6A14 shows the most broad substrate selectivity encompassing all essential amino acids as well as glutamine (Fig. 4a) and is upregulated in several cancers of epithelial origin, such as colon cancer^27^, cervical cancer^28^, and certain subtypes of breast cancer^29^.

The system L-type (leucine-preferring) amino acid transporter LAT1 is an amino acid antiporter, mediating the influx of BCAAs and bulky amino acids (phenylalanine, methionine, histidine, tryptophan, and tyrosine) into cells^116,117^ in exchange for efflux of intracellular substrates (EAA or glutamine) (Fig. 4a)^118,119^. The expression of *SLC7A5*, which encodes LAT1, is elevated in various cancers, including bladder, breast, cervical, and skin cancer, through the action of oncogenes and miRNAs. Hypoxia-induced factor 2α (HIF2α) and oncogenic c-MYC transactivate *SLC7A5*, whereas miR-126 inhibits *SLC7A5* expression^120,121^. The functional role of LAT1 in cancer, at least in part, is its ability to take in leucine with high affinity. Leucine is a well-known activator of mTOR signaling^122,123^ (Fig. 4a), and pharmacologic inhibition of LAT1 suppresses mTOR signaling and tumor growth (e.g., non-small cell lung cancer, oral cancer)^124,125^. LAT1-mediated leucine influx is coupled with another amino acid antiporter, ASCT2, which is encoded by *SLC1A5* (Fig. 4a)^126^. ASCT2 mediates the Na^+^-coupled influx of neutral amino acids (alanine, serine, cysteine, and glutamine) in mandatory exchange for the Na^+^-coupled efflux of other amino acids^126^. In the functional coupling between LAT1 and ASCT2, glutamine enters the cancer cell through ASCT2, which then effluxes out of the cell via LAT1 coupled to the entry of leucine^115^ (Fig. 4a). Consequently, genetic or pharmacological inhibition of ASCT2 impedes LAT1-mediated leucine uptake, leading to inactivation of mTOR signaling in cancer cells^127,128^. Notably, *SLC1A5* is also a target for c-MYC^111,112^, implying that cancer cells induce the two transporters (LAT1 and ASCT2) in a coordinated manner to enhance the functional coupling necessary for proliferation. *SLC1A5* expression is decreased by the tumor suppressor RB, supporting its role in cancer growth^129^. The amino acid transport system x−_c_ (xCT), which is encoded by *SLC7A11*, imports cystine and exports glutamate (Fig. 4a). The primary role of xCT is to provide cells with cysteine for glutathione synthesis. Similar to LAT1′s functional coupling with ASCT2, xCT appears to be functionally coupled to glutamine transporters^36^. In order to maintain glutathione levels, optimal xCT function requires intracellular glutamate pools to exchange with extracellular cysteine. Glutamine import from the extracellular environment, likely via ASCT2, can provide those glutamate pools through the GLS reaction. In addition to cysteine influx, xCT-mediated glutamate efflux also contributes to tumor aggressiveness by impacting the tumor microenvironment. Glutamate secreted to the extracellular environment via xCT causes peritumoral excitotoxic neuronal cell loss, creating room for tumor cell expansion^130^. Glutamate released through xCT can also act on tumor cells and promote their growth. In glioma, the secreted glutamate can activate AMPA receptors in both an autocrine and paracrine manner and cause intracellular Ca^2+^ oscillations, leading to tumor invasion^131^. In triple-negative breast cancer (TNBC), xCT-mediated glutamate release inhibits xCT and leads to intracellular cysteine depletion. In the absence of cysteine, EglN1, the main prolyl hydroxylase for the HIFα subunit, undergoes oxidative self-inactivation, resulting in HIF1α accumulation and tumor growth^132^. The TNBC study suggests that blocking xCT in TNBC can aggravate the disease, and thus, caution should be taken when targeting xCT. This notion is further supported by the findings that *Slc7a11*-null mice exhibit increased sensitivity to chemically induced carcinogenesis^133^. Basic amino acids, arginine and lysine, are transported via CAT1 and CAT2B, which are encoded by *SLC7A1* and *SLC7A2*, respectively (Fig. 4a). In a cancer setting, CAT1′s ability to transport arginine, rather than lysine, appears to be more relevant to tumor growth and survival. CAT1 suppression reduces arginine uptake and NO production, resulting in apoptotic cell death in breast cancer cells^134^.

Unlike essential amino acids whose source is solely the extracellular environment, nonessential amino acids can be produced, in most cases, through transamination reactions, which transfer the amino group from glutamate to a sugar precursor and generate α-KG. Aspartate aminotransferase (AST, also known as glutamic oxaloacetic transaminase (GOT), GOT1 in the cytosol and GOT2 in the mitochondria) generates aspartate from oxaloacetate and glutamate (Fig. 4b). Interestingly, recent studies have discovered an important role for aspartate synthesis in maintaining reducing potential. GOT1 consumes aspartate in the cytosol and transfers electrons into the mitochondria, which are accepted by the electron transport chain (ETC) and consume nicotinamide adenine dinucleotide (NADH) to regenerate NAD+^135,136^ (Fig. 4b). NAD+ can then be utilized for OAA generation and aspartate biosynthesis^135,136^. In PDAC, GOT1-derived oxaloacetate (OAA) fuels the TCA cycle, which is further converted to malate and pyruvate to produce NADPH from NADP+ to maintain the cellular redox state^25^. Pathophysiologically, GOT is closely related to alanine aminotransferase (ALT). ALT generates alanine from pyruvate and the nitrogen of glutamate. Under normal physiology, the AST (GOT)/ALT ratio is <1, but upon liver damage, including hepatocellular carcinoma (HCC), AST levels become higher than ALT (AST/ALT ratio>1). In addition to serving as a liver damage marker, ALT has implications for tumor growth. Inhibition of ALT induces oxidative phosphorylation and a subsequent increase in mitochondrial ROS, suggesting ALT as a putative target to promote mitochondrial metabolism and inhibit tumor growth^137^. A transaminase for serine synthesis is phosphoserine aminotransferase 1 (PSAT1) (Fig. 4b). It transfers nitrogen from glutamate to 3-phosphohydroxypyruvate to make phosphoserine. Similar to other transaminases, PSAT1 is associated with tumor aggressiveness, especially in breast cancer. Both the mRNA and protein levels of PSAT1 in ER-positive primary tumors are associated with poor clinical outcomes following tamoxifen treatment, suggesting that combination with a regimen targeting PSAT1 might enable therapeutic efficacy in this subset of breast cancer^26^.

Some amino acids are produced by non-transaminase reactions. The best-known non-transaminase NEAA-synthesizing enzyme would be glutaminase (GLS). This amidohydrolase generates glutamate and ammonia from glutamine. GLS activity has been shown to be critical for most cancer cell growth, at least in monolayer culture^138,139^. Because glutamate serves as a nitrogen donor for transaminase reactions, inhibition of GLS can potentially impact NEAA synthesis. Glutamine and asparagine are synthesized by amidation reactions (Fig. 2e). Glutamine synthetase (GS) ligates ammonia to glutamate and produces glutamine, whereas asparagine synthetase (ASNS) generates asparagine from aspartate and the amide nitrogen of glutamine (Fig. 4b). Asparagine synthesis is important for acquiring a tolerance to nutrient deficiency. It is enhanced upon glucose deprivation via induction of ASNS expression in PDAC^140^, and inhibition of ASNS leads to glutamine-withdrawal-induced cell death^48^. Glycine is synthesized from serine by the SHMT reaction; SHMT transfers a one-carbon unit from serine to THF and generates 5,10-meTHF.

In addition to amino acid transporter-mediated influx or biosynthetic pathways, macropinocytosis and proteolytic degradation of extracellular proteins can serve as a source of amino acids^52^. Inhibition of these processes impairs tumor growth, especially in KRAS-mediated PDAC, which uses macropinocytosed protein as a nutrient source. A recent study uncovered SLC38A9 as the mediator required to release essential amino acids from lysosomes, including leucine (Fig. 4a). PDAC utilizes SLC38A9, an arginine-sensing lysosomal protein with homology to amino acid transporters, to enable leucine generated via lysosomal proteolysis to exit lysosomes and activate mTORC1 and drive cell growth^141^. Collectively, these cancer cell dependencies on amino acids point to potential vulnerabilities that could be exploited in the design of anticancer therapies.

## Therapeutic applications from amino acid metabolism

A large body of work has demonstrated the diverse and important roles of amino acids in cancer metabolism^30,142^. Since the initial studies demonstrating the critical role of glutamine in the pathology of cancer, the field has continued to uncover the significance of other amino acids in metabolic alterations in cancer, and many efforts have been devoted to developing therapeutic agents targeting amino acid transport and catabolic/biosynthetic pathways. Although some amino acid-degrading enzymes, such as asparaginase and PEGylated arginine deiminase, have already been used in the clinic to treat tumors (asparaginase) and show antitumor efficacy in human patients (arginase), additional therapeutic targeting of cancer metabolism has led to surprisingly few new drugs^31–34^. In this section, we highlight a few promising targets in amino acid metabolism (Table 1).

**Table 1:** Select agents targeting amino acid metabolism that are developed, in trials or in the clinic, for the treatment of cancer.

Enzyme/ transporter	Inhibitor	Phase (status)	Cancer type
IDO1	BMS-986205 Navoximod (NLG-919) Pembrolizumab (in combination trial with epacadostat) PF-06840003	Phase I/II Phase I Phase II/III Phase II/III	Advanced cancer, melanoma, NSCLC Solid tumors RCC, melanoma, head and neck, gastro, malignant solid tumor, NSCLC, neoplasma (Phase I) Oligodendroglioma, astrocytoma, malignant glioma
IDO and TDO	HTI-1090 (dual inhibitor)	Phase I	Advanced solid tumors
GLS	CB-839 + cabozantinib CB-839 + talazoparib CB-839 + nivolumab CB-839 hydrochloride + osimertinib CB-839 + panitumumab, irinoteca hydrochloride CB-839 + azacytidine CB-839 + capecitabine CB-839 + palbociclib	Phase II Phase Ib/II Phase I/II Phase I/II Phase I/II Phase I/II Phase I/II Phase Ib/II	Renal cell carcinoma Solid tumors Melanoma, ccRCC, NSCLC Mutated stage iv NSCLC Metastatic and refractory RAS wildtype colorectal Advanced myelodysplastic syndrome Advanced solid tumors, colorectal cancer Solid tumors
ASCT2	V-9302		In vivo mouse models and in vitro
xCT	Sorafenib PRLX 93936 (erastin analog)	In clinic Phase I	Kidney, liver, and thyroid cancer Solid tumors
PHGDH	NCT-502 NCT-503		In vitro In vitro and mouse
GOT/AST	Aspulvinone O		Mouse
LAT1	JPH203		In vitro

Among the 20 canonical amino acids, glutamine metabolism has been a focal point in cancer therapy given cancer cells’ overreliance on glutamine. Both glutamine uptake and glutaminase activity have been actively investigated as oncological targets. Glutaminase inhibitors have been shown to reduce tumor burden^45,143,144^, and among the inhibitors, CB-839, the most advanced, is in clinical trials for multiple cancer types as a combination therapy. The glutamine (neutral amino acid) transporter ASCT2 is also an attractive target. The recently discovered V-9302, a selective inhibitor of ASCT2, showed in vivo antitumor efficacy, demonstrating the utility of a pharmacological inhibitor of glutamine transport in oncology^35^. Approaches targeting glutamate metabolism have been explored in several tumor models^145,146^. Glutamate conversion into α-KG through glutamate dehydrogenase (GDH) supports cancer cell growth in two ways: providing nitrogen for NEAA biosynthesis as well as an anaplerotic intermediate for the TCA cycle. Several inhibitors for GDH are available for experimental use and have been shown to inhibit tumor growth^145–147^.

Cysteine uptake plays an important role in cancer by maintaining redox balance, and numerous studies have shown the efficacy of xCT inhibition on tumor growth^36,148,149^. Importantly, a recent study revealed that xCT suppression acts synergistically with the immunotherapeutic agent anti-CTLA-4^150^. This study laid the groundwork for the clinical utilization of specific xCT inhibitors to expand the efficacy of existing anticancer immunotherapeutics^150^. Efforts have been made to target serine metabolism, with a specific focus on phosphoglycerate dehydrogenase (PHGDH), a rate-limiting enzyme in the serine de novo synthesis pathway^38,151–153^. Inhibition of PHGDH as a single agent, however, appears to only be effective under conditions of low serine availability^154^. In addition to the inhibition of serine biosynthesis, dietary restriction of serine and glycine has been explored in mice and was shown to be effective at limiting tumor growth in certain conditions where de novo serine synthesis activity is low^21,154^. Given the complex interplay between serine biosynthesis and environmental serine availability, selecting the proper tumor types would be critical for successful targeting of serine metabolism as a method of therapeutic intervention.

Manipulation of aspartate availability by dietary restriction or chemical inhibition is an emerging form of therapy. Initial high-throughput compound screening and further medicinal chemistry-based optimization developed two novel series of phenylurea-based compounds inhibiting GOT1 enzymatic activity with potencies in the low micromolar IC_50_ range^155^. This work lays the foundation for the development of potential therapeutics for the treatment of tumors that are highly dependent upon metabolic pathways involving GOT1 for the maintenance of redox homeostasis and sustained proliferation (e.g., PDAC).

In essential amino acid metabolism, tryptophan catabolism has been in the spotlight for cancer immunotherapy. Kynurenine produced from tryptophan by IDO1 and TDO2 binds to and activates the transcription factor aryl hydrocarbon receptor (AhR)^12–14^. AhR activation then leads to the generation of immune-tolerant DCs and regulatory T cells^15^, which foster a tumor immunological microenvironment that is defective in recognizing and eradicating cancer cells. Multiple IDO1 inhibitors have been actively evaluated in clinical trials^39^, and importantly, combination treatment of IDO inhibitor with the PD-1 immune checkpoint inhibitor pembrolizumab shows durable responses^40^. Although it is still in the early stages of development, targeting the IDO1/TDO2–KYN–AhR signaling pathway would potentially open new avenues for developing cancer immunotherapies.

BCAA metabolism has also received notable attention as a drug target. The BCAA catabolic enzyme BCAT1 has emerged as a useful prognostic cancer marker^156–158^. In glioblastoma, BCAA catabolism is upregulated, and BCATs are required for their growth^156^. The fact that *Bcat1*-knockout mice are viable^159,160^, suggests that there may be a good therapeutic window for targeting BCAT1-dependent tumors^161^. Inhibiting BCAA uptake also seems to be beneficial in certain tumors with high LAT1 expression (e.g., glioma)^162^. Indeed, in vitro screening has found a highly selective compound for LAT1, JPH203, which shows in vivo efficacy in reducing tumor growth^163^. When selecting patients for therapies targeting BCAA metabolism, however, tumor heterogeneity should be considered. PDAC shows decreased utilization of circulating BCAAs, and BCAA catabolism is dispensable for PDAC growth^161^. While BCAA catabolism is upregulated in glioblastoma and NSCLC, leukemia displays increased reverse reaction and generates BCAAs from branched-chain ketoacids (BCKAs)^161^, which supports the disease progression^164^. In liver cancer, BCAA supplementation has been shown to improve patient prognosis^165–167^. Understanding how the tissue-of-origin and surrounding environment interact and influence metabolic requirements in cancer will be critical to selecting the appropriate therapeutic regimens for patients.

## Concluding remarks

Studies from the past decades have proven the important role of amino acids in cancer metabolism in both a tumorigenic and tumor-suppressive way. Amino acids are involved in pathways that feed cancer cells and provide building blocks for cancer cell growth. The TCA cycle highlights an important mechanistic example of amino acid involvement supporting cancer. Amino acids such as glutamate, BCAAs, and threonine fuel the TCA cycle intermediates, resulting in the release of ATP and providing the required energy for oncogenic activities. Nucleotides, a critical building material for growth in normal and cancer cells, require amino acids as nitrogen and/or one-carbon donors (as a form of formate) for their biosynthesis. Some amino acids can regulate lipid biosynthesis by filling the acetyl-CoA pool or altering lipogenic gene expression. Amino acids also influence ROS homeostasis and epigenetic regulation through methylation and acetylation, all of which can enhance tumor aggressiveness. On the other hand, certain metabolic intermediates from amino acids can contribute to both tumorigenic and anti-tumorigenic activities. Nitric oxide (NO), a product of arginine metabolism (citrulline–NO pathway), can support tumor growth by promoting angiogenesis, but it can also act as a tumor suppressor, at least in part, by upregulating p53^78^.

Inhibition of amino acid metabolism is an active area of study in cancer metabolism; the field has yielded much success for cancer medications in vitro, but still faces many challenges to realize them in vivo. Some drugs targeting amino acid metabolism have been applied in a clinical setting and highlight the therapeutic potential of this mode of inhibition. However, a few things must be considered when targeting proteins involved in amino acid metabolism. Inhibition of the enzymes and/or transporters, especially related to EAA metabolism, is likely to be systemically toxic^168^ because of their physiological functions in normal tissues. Inhibition of NEAA metabolism, on the other hand, may not show full therapeutic efficacy in vivo. Although GLS inhibition is effective in hematologic and some solid cancers, PDAC displays adaptive metabolic networks that sustain proliferation during GLS inhibition and rarely shows any beneficial therapeutic effects. Thus, in the case of PDAC, targeting glutamine metabolism while taking into account these adaptive responses may yield clinical benefits for patients. In the same vein, PHGDH inhibition is not always sufficient to inhibit tumor growth^169,170^. Tumors arising in serine-limited environments can acquire a fitness advantage by upregulating serine synthesis pathway enzymes, implying that inhibition of serine metabolism can only be achieved by both PHGDH inhibition and dietary restriction of serine. Inhibiting amino acid transporters reduces amino acid availability to cells and thus depresses their function. However, targeting transporters might be challenging because of their broad specificity. It will be interesting to see if the newly developed LAT1 selective inhibitor shows in vivo efficacy^171^.

Because most metabolic inhibitors are unlikely to be effective cancer therapies as single agents, combination therapy is likely the best approach. Indeed, several studies have shown promising results using combination treatment of immunotherapeutic agents with metabolic inhibitors, for example, anti-CTLA-4 inhibitor with xCT inhibition^150^ and the PD-1 immune checkpoint inhibitor pembrolizumab with IDO1 inhibition^40,172^. All combinations show robust results in vitro and high potential to develop into treatment in vivo.

By fully understanding the metabolic flexibility and diversity of amino acid usage in cancer cells, it is possible to provide further insight into metabolic dependences and liabilities that can be exploited therapeutically.
